# Biallelic Truncating Variants in *SCN3B* Encoding Nav Channel Subunit β3 Lead to Neurodevelopmental Phenotype with and without Epilepsy and Ataxia

**DOI:** 10.1002/ana.78014

**Published:** 2025-08-23

**Authors:** Nathan Routledge, Maxime Lammens, Reza Maroofian, Bakht Beland, David Murphy, Asif Mir, Zia Ullah, Javeria Reza Alvi, Tipu Sultan, Stephanie Efthymiou, Frank Bosmans, Henry Houlden

**Affiliations:** ^1^ Department of Neuromuscular Disorders, Queen Square Institute of Neurology University College London London United Kingdom; ^2^ Molecular Physiology and Neurophysics Group, Department of Basic and Applied Medical Sciences Ghent University Ghent Belgium; ^3^ Experimental Pharmacology Group, Faculty of Medicine and Pharmaceutical Sciences Vrije Universiteit Brussel Brussels Belgium; ^4^ Department of Biological Sciences International Islamic University (IIU) Islamabad Pakistan; ^5^ Timergara Teaching Hospital Timergara Pakistan; ^6^ Department of Paediatric Neurology Institute of Child Health, Children's Hospital Lahore Lahore Pakistan

## Abstract

*SCN3B* encodes the β3 auxiliary subunit, essential for voltage‐gated Na^+^ (Nav) channel trafficking and gating. Although *SCN3B* has been associated with cardiac disorders, a link with neurodevelopmental disorders (NDD) has not been established. Using a genotype‐first approach, we identified homozygous truncating variants (c.281G>A‐β3^W94*^, c.584 + 1G>A‐β3^S196*^) in 2 consanguineous Pakistani families, leading to global developmental delay, intellectual disability and autism, with severe cognitive impairment, ataxia, and seizures in the case of β3^W94*^. Electrophysiological analysis revealed subtype‐specific gating alterations on multiple brain Nav channel subtypes. This is the first report linking *SCN3B* mutations to NDD, expanding our understanding of Nav channelopathies. ANN NEUROL 2025;98:864–870

Voltage‐gated Na^+^ (Nav) channel β‐subunits (*SCN1B*–*SCN4B*) play an important role in ion channel function by facilitating pore‐forming α‐subunit (*SCN1A*–*SCN5A*; *SCN8A*–*SCN11A*) trafficking to the cell surface and modulating gating mechanisms.^1^ Monoallelic variants in β‐subunit genes have been implicated in cardiac arrhythmias and cardiac channelopathies. However, among these, *SCN1B* is the only β‐subunit previously linked to neurodevelopmental disorders (NDD) and epilepsy, with both dominant and recessive pathogenic forms (OMIM: 600235).

*SCN3B* encodes the β3 auxiliary subunit that influences Nav channel function in both the nervous and cardiac systems.^2^ Although *SCN3B* variants have been primarily associated with cardiac disorders, such as Brugada syndrome, its role in neuronal excitability remains virtually unexplored.^3^, ^4^ Notably, *SCN3B* has been shown to affect the steady‐state inactivation or channel availability properties of Nav1.2,^2^ a subtype implicated in autism and developmental delay.^5^, ^6^ However, to date, no pathogenic *SCN3B* variants have been linked to NDD in humans.

In this study, we identified homozygous truncating *SCN3B* variants in 2 unrelated consanguineous families with neurodevelopmental phenotype, providing the first evidence of *SCN3B*‐related neurodevelopmental disorder and expanding the spectrum of Nav channelopathies.

## Methods

### Patient Recruitment, Clinical, and Genetic Investigation

**FIGURE 1:**
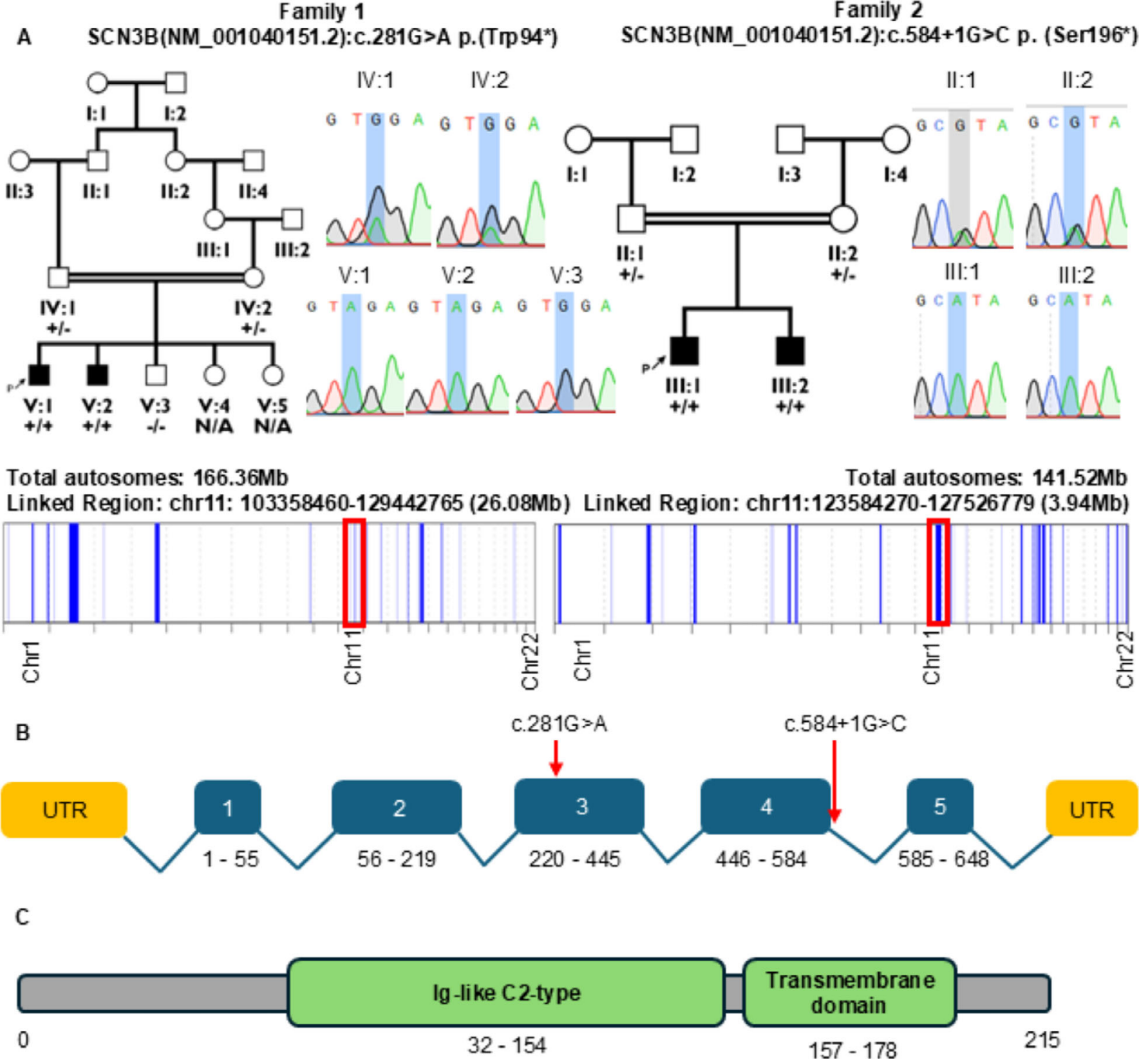
Genetic analysis overview of family 1 and 2. (A) Multigenerational pedigree of probands' families. + Wild‐type – Variant N/A indicates not available for sanger sequencing. Sangers sequence shown to confirm the segregation. (B) Schematic drawing of the longest *SCN3B* transcript (NM_001040151.2) consisting of 648 nucleotides in 5 exons. Variant position indicated by a red arrow. (C) Location of the variants c.281G>A, p.(Trp94*) and c.584 + 1G>A, p(Ser196*) in the SCN3B protein (ENSP00000299333.3; SCN3B‐201) consisting of 215 amino acids, X sign symbolizes where the truncation occurs. [Color figure can be viewed at www.annalsofneurology.org]

We applied a genotype‐first approach to screen Nav channel‐encoding genes in the University College London (UCL) Queen Square Genomics database, which includes over 40,000 exomes from a multi‐ethnic cohort with pediatric and adult‐onset neurological disorders and NDD. We identified and evaluated 2 independent consanguineous Pakistani families (Fig 1A). Ethical approval was granted by the Research Ethics Committee, Institute of Neurology, UCL (07/Q0512/26), and written informed consent was obtained in accordance with the Declaration of Helsinki. Comprehensive clinical evaluations and family histories were recorded for all affected individuals. Research exome sequencing, homozygosity mapping, and Sanger segregation analysis were conducted at the UCL Queen Square Neurogenetics Lab, following established protocols.^7^

### Functional Assays

C‐terminal fragments for β3^W94^* and β3^S196*^ were constructed (Genscript, USA) and subcloned into the human β3 clone (Origene, NM_018400.3) using restriction enzymes (New England Biolabs, USA). Correct cloning was confirmed by Sanger sequencing (Eurofins Genomics, Germany). For transient expression in CHO‐K1 cells, Lipofectamine LTX (Invitrogen, USA) was used in combination with 500ng complementary DNA (cDNA) following the manufacturer's protocol. After 48 hours, cells were stained for *SCN3B*, F‐actin, and nuclei (Supporting Information methods), and confocal imaging was performed using a Leica TCS SP8 X microscope (40×/1.30 oil objective). Images were processed with ImageJ (NIH, USA).

For electrophysiological testing, sequences of human Nav1.1 (Origene, NM_001165963.1), Nav1.2 (Origene, NM_021007.2), Nav1.3 (Origene, NM_006922.2), and Nav1.6 (Origene, NM_014191.3) were confirmed by automated Sanger sequencing. RNA of these and wild‐type (WT)/mutant β3 constructs was synthesized using T7 polymerase (Invitrogen, USA) after linearizing the cDNA with an appropriate restriction enzyme. Nav channel and β3 RNA were microinjected into defolliculated *Xenopus laevis* oocytes (Nasco, USA) in a 1:10 molar ratio and incubated for 1 to 4 days at 17°C in Barth's medium supplemented with 50μg/ml gentamycin, pH 7.4 with NaOH (Sigma, USA). Electrophysiological characteristics were studied using the 2‐electrode voltage‐clamp technique (OC‐725C; Warner Instruments, USA) as previously described.^8^ Statistical differences were tested with 1‐way analysis of variance (ANOVA) or Kruskal‐Wallis tests and corrected for multiple testing using Prism 10 (GraphPad, USA).

## Results

### Identification of a Homozygous *SCN3B* Splice Site Variant and a Homozygous Nonsense Variant in Siblings in 2 Families

Genotype‐first screening of exome data led to the identification of 2 homozygous *SCN3B* variants within regions of homozygosity (AutoMap): a nonsense substitution (NM_001040151.2: c.281G>A; p.(Trp94*); β3^W94^*) in 2 brothers from family 1 (Pakistan) and a splice donor variant (NM_001040151.2: c.584 + 1G>A; β3^S196*^) in 2 sisters from family 2 (Pakistan) (Fig 1A). Sanger sequencing confirmed segregation of the variants within the families. We also screened multiple independent sequencing databases and data‐sharing platforms, including GeneMatcher, but were unable to identify any additional families. The nonsense variant (c.281G>A; p.(Trp94Ter)) was novel, absent in both heterozygous and homozygous forms in Genome Aggregation Database (gnomAD), while the splice variant (c.584 + 1G>A) had an extremely rare heterozygous allele frequency of 0.00000137 in gnomAD.

### Clinical Findings

Family 1 (β3^W94^*) consists of 2 affected brothers and 3 unaffected siblings, born to consanguineous Pakistani parents (Table 1). The elder brother (11 years old) presented with global developmental delay, hypotonia, autistic traits, severe intellectual disability, irritability, and aggressiveness. He has never attained speech or independent walking and has had monthly generalized tonic–clonic seizures for 5 months, well controlled with sodium valproate. Examination revealed macrotia, reduced deep tendon reflexes, and ataxia. Electroencephalography (EEG) showed generalized epileptiform discharges, while magnetic resonance imaging (MRI) was unremarkable, with no signs of cranial nerve involvement or regression. The younger brother (6 years, 9 months old) had a similar phenotype, with global developmental delay, hypotonia, autistic traits, severe intellectual disability, and aggressiveness. He has never developed speech or independent walking and has had monthly generalized tonic–clonic seizures for 3 months, also controlled with sodium valproate. Findings were like his brother's, including, weak muscle strength, ataxia, and reduced reflexes, with EEG showing generalized epileptiform discharges and no MRI abnormalities. Macrotia was also seen as an incidental finding in the siblings.

**TABLE 1:** Summary of Genetic and Clinical Features of Cases with *SCN3B*‐Related Neurodevelopmental Disorders

Family‐Patient	F1 – V:1	F1 – V:2	F2 – III:1	F2 – III:2
cDNA change	c.281G>A	c.281G>A	c.584 + 1G>A	c.584 + 1G>A
Protein change	p.Trp94Ter	p.Trp94Ter	p.Ser196Ter	p.Ser196Ter
Zygosity	Homozygous	Homozygous	Homozygous	Homozygous
Age (sex)	11 yr (M)	6 yr (M)	9 yr (F)	7 yr (F)
Ethnic background	Pakistan	Pakistan	Pakistan	Pakistan
Hypotonia	+	+	−	−
GDD/ID	Severe	Severe	Mild	Severe
Microcephaly	−	−	−	−
Failure to thrive/short stature	−/−	−/−	−/−	−/−
Autism	+	+	+	+
Other behavioral and psychiatric features	Irritability, fussiness, inattentive, hyperactive, aggressiveness, sleep disturbance	Irritability, inattentive, sleep disturbance	Aggressiveness	Aggressiveness
Seizures (age of onset)	+ (5 mo)	+ (3 mo)	−	−
Seizure type	Generalized tonic clonic,	Generalized tonic clonic	−	−
Frequency of seizures	Monthly	Monthly	−	−
Frequency of seizures before and after therapy	Controlled with ASM	Controlled with ASM	−	−
Current ASM	Sodium valproate, clonazepam	Sodium valproate	−	−
Lifetime ASM	Sodium valproate	Sodium valproate	−	−
Febrile seizures	+	+	−	−
Duration of seizures	2–3 min	2–3 min	−	−
Status epilepticus	None	None	−	−
Clustering	+	+	−	−
EEG findings	Generalized epileptiform discharges	Generalized epileptiform discharges	−	−
Deep tendon reflexes	Reduced	Reduced	+2	Left brisk
Muscle tone	Normal	Hypotonia	Normal	Normal
Muscle weakness	+	+	−	−
Ataxia	+	+	−	−
Dyskinetic movements	Stereotypical	Stereotypical	Stereotypical	Stereotypical
Gait abnormalities	Broad based	Non ambulatory	−	Limping
Brain MRI abnormalities (age)	No (5 yr)	Not done	Not done	No
Dysmorphism	Macrotia	Macrotia	−	−
Cardiovascular problems	−	−	−	−
Bowel and urinary incontinence	+	+	+	+
Skeletal/orthopedic findings	−	−	−	Hyperlaxity

+ indicates has feature, − indicates does not have feature.

ASM = Anti‐seizure medication; cDNA = complementary DNA; EEG = electroencephalography; F = female; M = male; MRI = magnetic resonance imaging.

Family 2 (β3^S196*^) consists of 2 affected sisters, born to consanguineous Pakistani parents (Table 1). The elder sister (9 years old) presented with mild developmental delays, including delayed motor, speech, and cognitive milestones. She walked independently by 20 months, spoke in 4 to 5‐word sentences, and exhibited mild speech difficulties, autistic traits, mild intellectual disability, and aggressiveness. Neurological and visual examinations were normal, with no systemic symptoms. The younger sister (7 years old) had a more severe phenotype, with global developmental delay affecting speech, cognition, and motor milestones. She lacked comprehensible speech, walked at 24 months with a limping gait because of hip dislocation, and exhibited autistic traits, severe intellectual disability, and aggressiveness. She also had generalized hyperlaxity, leading to hip dislocation diagnosed in infancy, requiring 2 corrective surgeries. MRI and systemic evaluations were unremarkable. Both sisters were well nourished, with normal reflexes and no cranial nerve involvement.

### Loss of β3 Alters Nav Channel Gating

**FIGURE 2:**
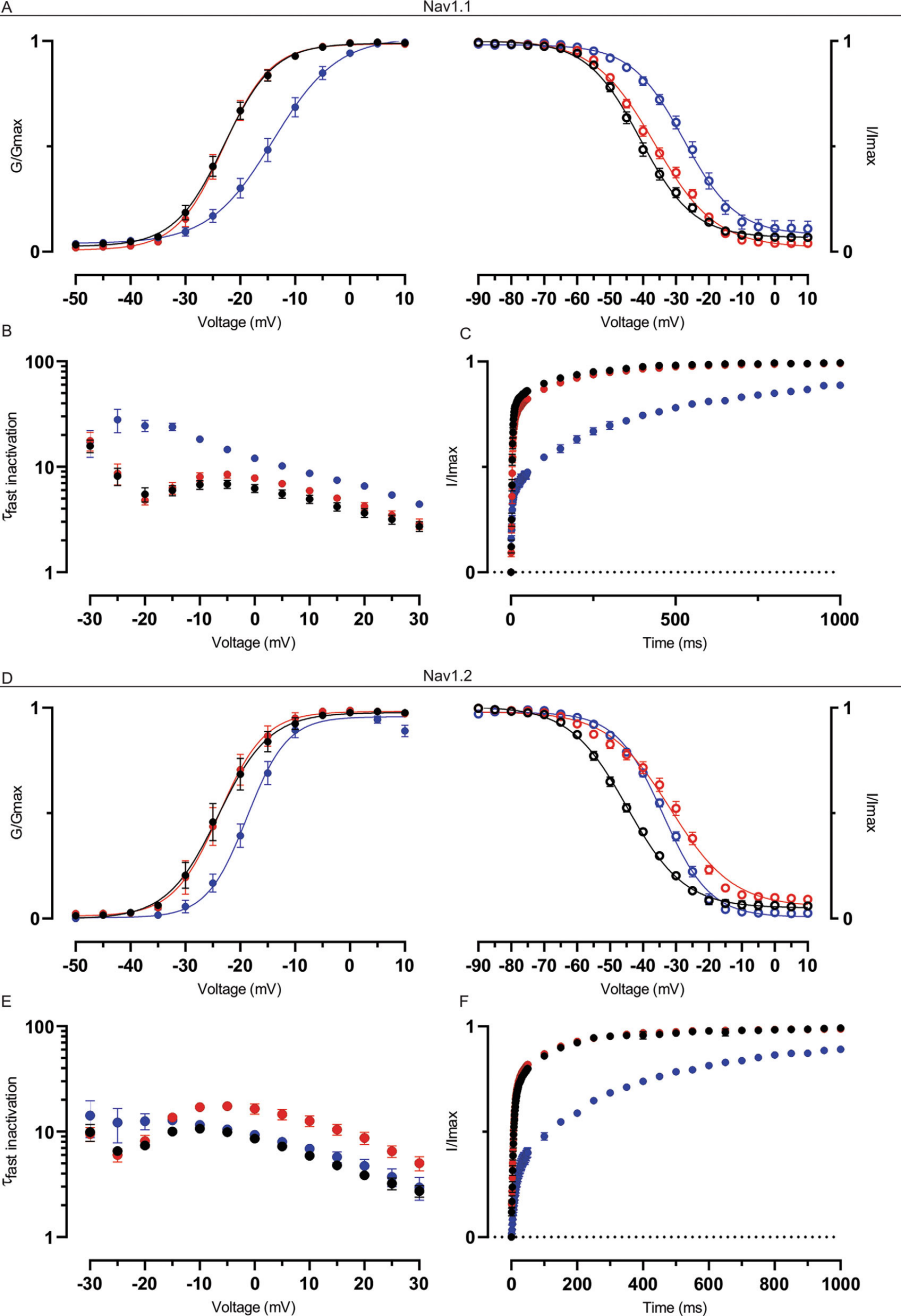
Effects of β3 mutants on Na_V_1.1 and Na_V_1.2. (A) Normalized conductance‐voltage (left) and channel availability (right) curves for Na_V_1.1 in the presence of wild‐type (WT) β3 (black), β3^S196*^ (red), or β3^W94^* (blue). (B) Inactivation time constants for Na_V_1.1 with WT β3 (black), β3^S196*^ (red), or β3^W94^* (blue). (C) Normalized recovery from fast inactivation for Na_V_1.1 with WT β3 (black), β3^S196*^ (red), or β3^W94^* (blue). (D) Normalized conductance‐voltage (left) and channel availability (right) curves for Na_V_1.2 in the presence of WT β3 (black), β3^S196*^ (red), or β3^W94^* (blue). (E) Inactivation time constants for Na_V_1.2 with WT β3 (black), β3^S196*^ (red), or β3^W94^* (blue). (F) Normalized recovery from fast inactivation for Na_V_1.2 with WT β3 (black), β3^S196*^ (red), or β3^W94^* (blue). All data is shown as mean ± standard error of the mean. [Color figure can be viewed at www.annalsofneurology.org]

We first examined the gating characteristics of 4 Nav channel subtypes predominantly expressed in the brain (Nav1.1, Nav1.2, Nav1.3, and Nav1.6) in the presence of β3^WT^ or β3^W94^*. The nonsense variant is located within the Ig‐like domain and is reasonably expected to result in a complete loss of function of *SCN3B* because of the loss of all functional hallmarks including the Ig region and transmembrane segment (see Fig 1C and Fig S1A).^9^, ^10^ Indeed, β3^W94^* abolished the effect of β3^WT^ on the conductance‐voltage (G‐V) relationship of Nav1.2, Nav1.3, and Nav1.6, while causing a significant depolarizing shift on Nav1.1 activation voltage (Table S1). Moreover, β3^W94^* depolarized channel availability of all tested channel subtypes (Fig 2A, D; Figs S2A, D, G, J and S3A, D; and Table S1), and significantly hampered Nav1.1 and Nav1.6 inactivation kinetics (see Fig 2B, E; Figs S2B, E, H, K, and S3B, E). Last, β3^W94^* also reduced recovery from inactivation of all tested channels (see Fig 2C, F and Fig S2C, F) compared to β3^WT^. Overall, β3^W94^* largely phenocopied the absence of β3, which is consistent with a functional null effect.

### β3^S196^ * Alters Na_V_1.2 Gating

β3^S196*^ harbors a premature stop codon producing a truncated protein lacking the final 20 amino acids of the intracellular C‐tail (see Fig 1C and Fig S1A), a region that may play a critical role in trafficking and subcellular localization of both the β‐subunit^11^, ^12^ and associated Nav channels.^13^ Therefore, we compared cell surface expression of β3^WT^ and β3^S196*^ in CHO‐K1 cells via transient transfection and immunocytochemistry. Results demonstrate that β3^S196*^ localizes to the plasma membrane similarly to the WT protein (Fig S1B, C), indicating that C‐tail deletion does not impair membrane trafficking.

To assess whether C‐terminal truncation of β3 affects Nav channel gating, we expressed both partners together and measured potential changes in gating parameters. In contrast to β3^W94^*, β3^S196*^ produces distinct, Nav channel subtype‐specific effects. Although β3^S196*^ preserved its ability to modulate the G‐V relationship of all tested channels, its effect on channel availability of Nav1.2 was abolished (see Fig 2D and Table S1), leading to an increased pool of available Nav1.2 channels at resting membrane potential. For Nav1.1, truncation resulted in a small, but significant depolarizing shift in channel availability (Fig 2A and Table S1). Furthermore, β3^S196*^ significantly prolonged inactivation time constants of Nav1.2 at voltages ranging from −10 to +15 mV, whereas Nav1.1 inactivation remained unaffected. β3^WT^ and β3^S196*^ facilitated recovery from inactivation of these channels to a similar extent (see Fig 2C, F). Nav1.3 and Nav1.6 gating properties remained unaffected, suggesting that these subtypes may be less dependent on the C‐tail of β3 for regulatory functions (Fig S2A–F and Table S1). Combined, these data suggest the possibility for increased excitability in neurons that express Nav1.2, and driven by β3^S196*^.

## Discussion

To date, β‐subunit genes have been primarily associated with cardiac phenotypes, also with only *SCN1B* being associated with NDD (Table S2). By leveraging a multi‐ethnic neurological exome database, we have identified 2 families with homozygous *SCN3B* variants presenting neurodevelopmental phenotypes. Electrophysiological analysis revealed that β3^S196*^ can alter Nav channel function in a subtype‐specific manner, most significantly affecting Nav1.2 inactivation, possibly leading to a gain‐of‐function effect in neurons that express this subtype. Interestingly, the C‐tail of β3 is known to interact with the C‐terminal domain of Nav channels,^14^ a domain that plays an important role in channel inactivation.^15^ Disruption of this interaction through deletion of the C‐terminal 20 amino acids in β3^S196*^ could, therefore, provide a possible disease mechanism. Moreover, given that Nav1.2 dysfunction is strongly associated with autism spectrum disorder (ASD) and neurodevelopmental delay,^5^, ^6^ these results provide a potential mechanistic link between *SCN3B* truncations and neurodevelopmental phenotypes. Furthermore, the complete loss‐of‐function β3 variant in family 1 resulted in a more severe phenotype, including severe intellectual disability with epilepsy and ataxia, which parallels the severe end of *SCN2A*‐related disorders (OMIM: 182390) as well as the loss of function of β3^WT^ effects on all other tested brain Nav channels. This suggests a genotype–phenotype correlation, where partial β3 dysfunction results in milder phenotypes, while complete loss of β3 leads to more severe neurodevelopmental outcomes, warranting further study in additional cases.^16^, ^17^

Previous studies using *SCN3B* knockout mice have shown that, in contrast to our findings, loss of β3 results in clear cardiac abnormalities without apparent neurological defects.^18^, ^19^ These studies, however, did not include any behavioral assays scoring autism or NDD‐related deficits. Moreover, although our results show a virtually complete loss of brain Nav channel modulation by β3^W94^*, we cannot rule out the expression of the truncated peptide in vivo possibly causing additional harmful effects contributing to NDD. Last, despite the absence of cardiac abnormalities in our patients, follow‐up is required to assess the development of these symptoms.

The identification of *SCN3B* as a neurodevelopmental gene underscores the power of a genotype‐first approach in uncovering novel disease associations.^20^ Rare neurological disorders often present with heterogeneous phenotypes, making traditional phenotype‐driven diagnoses challenging. By analyzing the entire family of Nav channels as well as related genes, we were able to identify new pathogenic connections that might have been missed with a single‐patient, phenotype‐driven approach. As such, our study supports the concept that investigating gene families with shared structural and functional properties can be an effective strategy for identifying ultra‐rare disease associations that might otherwise remain undiagnosed.^21^

Although *SCN3B* is ubiquitously expressed in the brain, direct association of mutations with ASD or neurodevelopmental delay remains unestablished. Here, for the first time, we identify homozygous truncating *SCN3B* variants associated with NDD and ASD. These findings expand the landscape of Nav channelopathies,^22^ reinforce the importance of β‐subunits in neuronal excitability, and suggest that *SCN3B* should be considered in the genetic evaluation of NDD, particularly in consanguineous families or cases with undiagnosed ion channel‐related phenotypes.

## Author Contributions

N.R., M.L., S.E., F.B., and H.H. contributed to the conception and design of the study; N.R., M.L., F.B., R.M., S.E., B.B., A.M., Z.U., J.R.A., T.S., and D.M., contributed to the acquisition and analysis of data; N.R., M.L., F.B., and R.M. contributed to drafting the text or preparing the figures.

## Potential Conflicts of Interest

None.

## Supporting information


**Data S1.** Supporting Information

## Data Availability

The data that support the findings of this study are available on request from the corresponding author. The data are not publicly available because of privacy or ethical restrictions.

## References

[1] Bouza AA , Isom LL . Voltage‐gated Sodium Channel β subunits and their related diseases. Handb Exp Pharmacol 2018;246:423–450. 10.1007/164_2017_48. 28965169 PMC6338345

[2] Morgan K , Stevens EB , Shah B , et al. Beta 3: an additional auxiliary subunit of the voltage‐sensitive sodium channel that modulates channel gating with distinct kinetics. Proc Natl Acad Sci U S A 2000;97:2308–2313. 10.1073/pnas.030362197. 10688874 PMC15797

[3] Peeters U , Scornik F , Riuró H , et al. Contribution of cardiac Sodium Channel β‐subunit variants to Brugada syndrome. Circ J 2015;79:2118–2129. 10.1253/circj.CJ-15-0164. 26179811

[4] Olesen MS , Jespersen T , Nielsen JB , et al. Mutations in sodium channel β‐subunit SCN3B are associated with early‐onset lone atrial fibrillation. Cardiovasc Res 2011;89:786–793. 10.1093/cvr/cvq348. 21051419

[5] Satterstrom FK , Kosmicki JA , Wang J , et al. Large‐scale exome sequencing study implicates both developmental and functional changes in the neurobiology of autism. Cell 2020;180:568–584.e523. 10.1016/j.cell.2019.12.036. 31981491 PMC7250485

[6] Sanders SJ , Murtha MT , Gupta AR , et al. De novo mutations revealed by whole‐exome sequencing are strongly associated with autism. Nature 2012;485:237–241. 22495306 10.1038/nature10945 PMC3667984

[7] Scala M , Efthymiou S , Sultan T , et al. Homozygous SCN1B variants causing early infantile epileptic encephalopathy 52 affect voltage‐gated sodium channel function. Epilepsia 2021;62:e82–e87. 10.1111/epi.16913. 33901312 PMC8585727

[8] de Cássia Collaço R , Lammens M , Blevins C , et al. Anxiety and dysautonomia symptoms in patients with a Na(V)1.7 mutation and the potential benefits of low‐dose short‐acting guanfacine. Clin Auton Res 2024;34:191–201. 10.1007/s10286-023-01004-1. 38064009 PMC11805752

[9] Noland CL , Chua HC , Kschonsak M , et al. Structure‐guided unlocking of NaX reveals a non‐selective tetrodotoxin‐sensitive cation channel. Nat Commun 2022;13:1416. 35301303 10.1038/s41467-022-28984-4 PMC8931054

[10] Namadurai S , Balasuriya D , Rajappa R , et al. Crystal structure and molecular imaging of the nav channel β3 subunit indicates a trimeric assembly. J Biol Chem 2014;289:10797–10811. 10.1074/jbc.M113.527994. 24567321 PMC4036194

[11] Malhotra JD , Thyagarajan V , Chen C , Isom LL . Tyrosine‐phosphorylated and nonphosphorylated sodium channel beta1 subunits are differentially localized in cardiac myocytes. J Biol Chem 2004;279:40748–40754. 10.1074/jbc.M407243200. 15272007

[12] Zhang ZN , Li Q , Liu C , et al. The voltage‐gated Na+ channel Nav1.8 contains an ER‐retention/retrieval signal antagonized by the beta3 subunit. J Cell Sci 2008;121:3243–3252. 10.1242/jcs.026856. 18782866

[13] Ishikawa T , Takahashi N , Ohno S , et al. Novel SCN3B mutation associated with brugada syndrome affects intracellular trafficking and function of Nav1.5. Circ J 2013;77:959–967. 10.1253/circj.cj-12-0995. 23257389

[14] Spampanato J , Kearney JA , de Haan G , et al. A novel epilepsy mutation in the Sodium Channel SCN1A identifies a cytoplasmic domain for β subunit interaction. J Neurosci 2004;24:10022–10034. 10.1523/jneurosci.2034-04.2004. 15525788 PMC6730248

[15] Mantegazza M , Yu FH , Catterall WA , Scheuer T . Role of the C‐terminal domain in inactivation of brain and cardiac sodium channels. Proc Natl Acad Sci 2001;98:15348–15353. 10.1073/pnas.211563298. 11742069 PMC65032

[16] Howell KB , McMahon JM , Carvill GL , et al. SCN2A encephalopathy: a major cause of epilepsy of infancy with migrating focal seizures. Neurology 2015;85:958–966. 10.1212/wnl.0000000000001926. 26291284 PMC4567464

[17] Rusina E , Simonti M , Duprat F , et al. Voltage‐gated sodium channels in genetic epilepsy: up and down of excitability. J Neurochem 2024;168:3872–3890. 10.1111/jnc.15947. 37654020 PMC11591406

[18] Hakim P , Brice N , Thresher R , et al. Scn3b knockout mice exhibit abnormal sino‐atrial and cardiac conduction properties. Acta Physiol 2010;198:47–59. 10.1111/j.1748-1716.2009.02048.x. PMC3763209 19796257

[19] Hakim P , Gurung IS , Pedersen TH , et al. Scn3b knockout mice exhibit abnormal ventricular electrophysiological properties. Prog Biophys Mol Biol 2008;98:251–266. 19351516 10.1016/j.pbiomolbio.2009.01.005 PMC2764399

[20] Wilczewski CM , Obasohan J , Paschall JE , et al. Genotype first: clinical genomics research through a reverse phenotyping approach. Am J Hum Genet 2023;110:3–12. 10.1016/j.ajhg.2022.12.004. 36608682 PMC9892776

[21] Taruscio D , Gahl WA . Rare diseases: challenges and opportunities for research and public health. Nat Rev Dis Primers 2024;10:13. 10.1038/s41572-024-00505-1. 38424095

[22] O'Malley HA , Isom LL . Sodium channel β subunits: emerging targets in channelopathies. Annu Rev Physiol 2015;77:481–504. 10.1146/annurev-physiol-021014-071846. 25668026 PMC4817109

