# Supplementary material for: Biallelic Truncating Variants in SCN3B Encoding Nav Channel Subunit β3 Lead to Neurodevelopmental Phenotype with and without Epilepsy and Ataxia
Source: Ann Neurol. 2025 Aug 23;98(4):864–70. doi: 10.1002/ana.78014 (PMC12542317; doi:10.1002/ana.78014)
Supplement: Supplementary file 1 — Data S1. Supporting Information [file ANA-98-864-s001.docx]

**Supplementary materials**

**Methods**

**Cell culture**

CHO-K1 Flp-In cells (Invitrogen, USA) were cultured in Ham’s 12 medium (Gibco, USA) supplemented with 10% (v/v) fetal bovine serum (Gibco, USA) and 1% (v/v) Penicillin/Streptomycin (Gibco, USA) at 37°C under 5% CO_2_.

**Immunocytochemistry**

After transfection, cells were incubated at 37°C for 24h followed by 24h at 28°C before seeding on poly-D-Lysine (PDL)-coated coverslips. Cells were washed in PBS, fixed in 4% paraformaldehyde (15 min, RT), and blocked with 10% Normal Goat Serum (Vector Laboratories, USA, 60 min, RT). To detect surface-expressed β3, the cells were stained with the primary mouse anti-*SCN3B* antibody (1:100, Novus Biologicals, UK) overnight at 4°C before membrane permeabilization (0.1% Triton X-100, Sigma-Aldrich, USA). Subsequently secondary staining was performed by incubating the coverslips with goat anti-mouse Alexa 488 (1:500, Invitrogen, USA) and rhodamine-phalloidin (1:40, Invitrogen, USA) for 1h at RT. After DAPI staining (1µg/ml, 5 min, Merck Life Science, Germany), coverslips were mounted in Prolong Gold Antifade (Invitrogen, USA).

**Tables and figures**

**Supplementary table S1: Gating parameters of brain Nav channel subtypes with and without β3 subunit variants.** All data is shown as mean ± SEM. *P < 0.05, **P < 0.01, ***P < 0.001, ****P < 0.0001; significance levels are only shown against WT β3 for clarity.

| **Condition** | **Activation** | | | **Steady state**  **inactivation** | | | **Number oocytes** |
| --- | --- | --- | --- | --- | --- | --- | --- |
|  | **V_1/2_ (mV)** | | **Slope** | **V_1/2_ (mV)** | | **Slope** |  |
| **Nav1.1 ± β3** | | | | | | | |
| Nav1.1 + β3 WT | -22.88 ± 0.93 | 4.50 ± 0.31 | | -40.72 ± 1.05 | -8.05 ± 0.34 | | 15 |
| Nav1.1 | -18.69 ± 0.53** | 4.17 ± 0.17 | | -29.01 ± 0.59**** | -6.80 ± 0.25 | | 15 |
| Nav1.1 + β3 W94Ter | -13.95 ± 1.26**** | 5.84 ± 0.51 | | -27.57 ± 1.09**** | -7.97 ± 0.52 | | 7 |
| Nav1.1 + β3 S196Ter | -22.93 ± 1.01 | 4.30 ± 0.33 | | -36.39 ± 0.98** | -9.27 ± 0.33 | | 13 |
| **Nav1.2 ± β3** | | | | | | | |
| Nav1.2 + β3 WT | -23.55 ± 1.56 | 4.67 ± 0.56 | | -45.00 ± 0.79 | -8.71 ± 0.34 | | 6 |
| Nav1.2 | -18.82 ± 1.47 | 4.41 ± 0.48 | | -32.89 ± 0.21**** | -7.08 ± 0.22 | | 6 |
| Nav1.2 + β3 W94Ter | -18.68 ± 0.96 | 3.67 ± 0.37 | | -33.86 ± 0.57**** | -7.09 ± 0.26 | | 8 |
| Nav1.2 + β3 S196Ter | -23.57 ± 1.66 | 4.15 ± 0.52 | | -31.75 ± 1.32**** | -9.49 ± 0.51 | | 6 |
| **Nav1.3 ± β3** | | | | | | | |
| Nav1.3 + β3 WT | -17.95 ± 0.66 | 4.74 ± 0.24 | | -34.31 ± 0.49 | -9.16 ± 0.22 | | 19 |
| Nav1.3 | -14.85 ± 0.59* | 4.68 ± 0.24 | | -31.15 ± 0.83** | -8.64 ± 0.35 | | 12 |
| Nav1.3 + β3 W94Ter | -15.76 ± 1.26 | 3.77 ± 0.44 | | -26.95 ± 1.24**** | -8.84 ± 0.41 | | 12 |
| Nav1.3 + β3 S196Ter | -18.59 ± 0.37 | 4.81 ± 0.15 | | -34.44 ± 0.53 | -9.35 ± 0.26 | | 20 |
| **Nav1.6 ± β3** | | | | | | | |
| Nav1.6 + β3 WT | -25.00 ± 1.38 | 5.10 ± 0.49 | | -56.45 ± 0.73 | -5.58 ± 0.22 | | 6 |
| Nav1.6 | -19.39 ± 0.94* | 5.17 ± 0.31 | | -50.49 ± 1.38 | -10.20 ± 0.58 | | 6 |
| Nav1.6 + β3 W94Ter | -16.29 ± 0.51**** | 5.68 ± 0.21 | | -44.99 ± 0.59*** | -10.44 ± 0.49 | | 9 |
| Nav1.6 + β3 S196Ter | -23.85 ± 1.60 | 4.69 ± 0.53 | | -55.67 ± 0.64 | -5.55 ± 0.16 | | 6 |

| Gene | Phenotype* | Inheritance |
| --- | --- | --- |
| SCN1B | Atrial fibrillation, familial, 13 | AD |
|  | Brugada syndrome 5 |  |
|  | Cardiac conduction defect, nonspecific |  |
|  | Developmental and epileptic encephalopathy 52 | AR |
|  | Generalized epilepsy with febrile seizures plus, type 1 | AD |
| SCN2B | Atrial fibrillation, familial, 14 | AD |
| SCN3B | Atrial fibrillation, familial, 16 | AD |
|  | Brugada syndrome 7 | AD |
| SCN4B | Atrial fibrillation, familial, 17 | AD |
|  | Long QT syndrome 10 | AD |

**Supplementary table S2: Overview of β-subunit genes and their associated disorders**

* From Online Mendelian Inheritance in Man (OMIM) database

AD = Autosomal Dominant AR = Autosomal Recessive

**
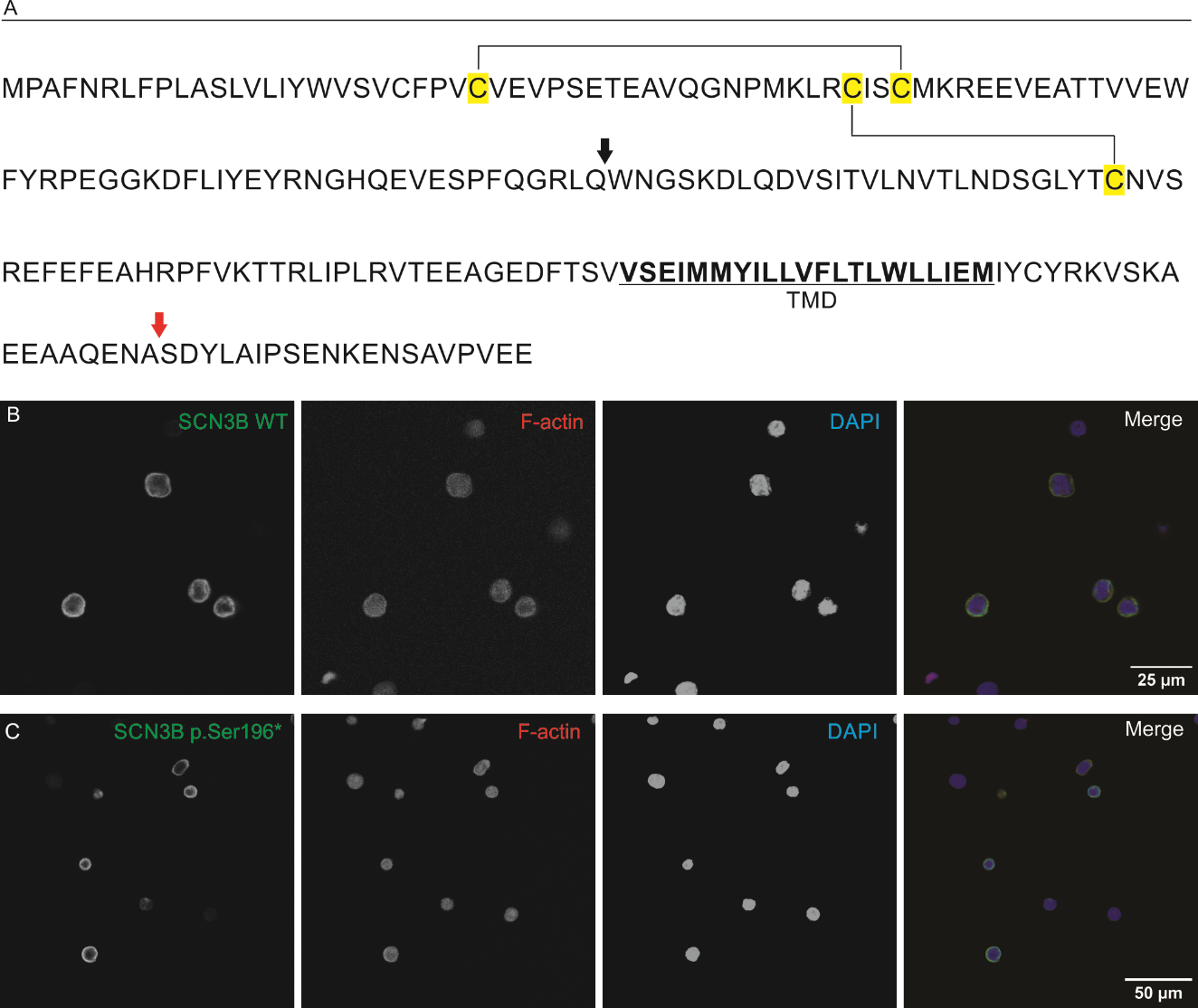
**

**Supplementary figure 1: Protein sequence and membrane trafficking of WT β3 and β3^S196*^.** (**A**) Protein sequence of WT β3 consisting of 215 amino acids. The transmembrane domain (TMD) is underlined and annotated in bold. Structurally important cysteine residues in the extracellular Ig-domain are indicated in yellow and vital cysteine bridges are shown by connecting lines. Arrows indicate sites of premature protein termination for β3^W94^* (black) and β3^S196*^ (red). **(B)** Representative image of CHO-K1 cells expressing WT *SCN3B*, showing localization in the plasma membrane following transient transfection. **(C)** Representative image of CHO-K1 cells expressing β3^S196*^, similarly localized to the plasma membrane. Cells were co-stained for F-actin (cytoskeleton) and DAPI (nucleus) to highlight cellular architecture.

**Supplementary figure 2: Effect of WT β3 on Na_V_1.1, Nav1.2, Nav1.3 and Na_V_1.6.** **(A)** Normalized conductance-voltage (left) and channel availability (right) curves for Na_V_1.1 in the absence (grey) and presence (black) of WT of β3. **(B)** Inactivation time constants for Na_V_1.1 without β3 (grey) and with WT β3 (black). **(C)** Normalized recovery from fast inactivation for Na_V_1.1 without β3 (grey) and with WT (black) β3. **(D)** Normalized conductance-voltage (left) and channel availability (right) curves for Na_V_1.2 in the absence (grey) and presence (black) of WT of β3. **(E)** Inactivation time constants for Na_V_1.2 without β3 (grey) and with WT (black) β3. **(F)** Normalized recovery from fast inactivation for Na_V_1.2 without β3 (grey) and with WT (black) β3. **(G)** Normalized conductance-voltage (left) and channel availability (right) curves for Na_V_1.3 in the absence (grey) and presence (black) of WT of β3. **(H)** Inactivation time constants for Na_V_1.3 without β3 (grey) and with WT β3 (black). **(I)** Normalized recovery from fast inactivation for Na_V_1.3 without β3 (grey) and with WT (black) β3. **(J)** Normalized conductance-voltage (left) and channel availability (right) curves for Na_V_1.6 in the absence (grey) and presence (black) of WT of β3. **(K)** Inactivation time constants for Na_V_1.6 without β3 (grey) and with WT β3 (black). **(L)** Normalized recovery from fast inactivation for Na_V_1.6 without β3 (grey) and with WT (black) β3. All data is shown as mean ± SEM.**
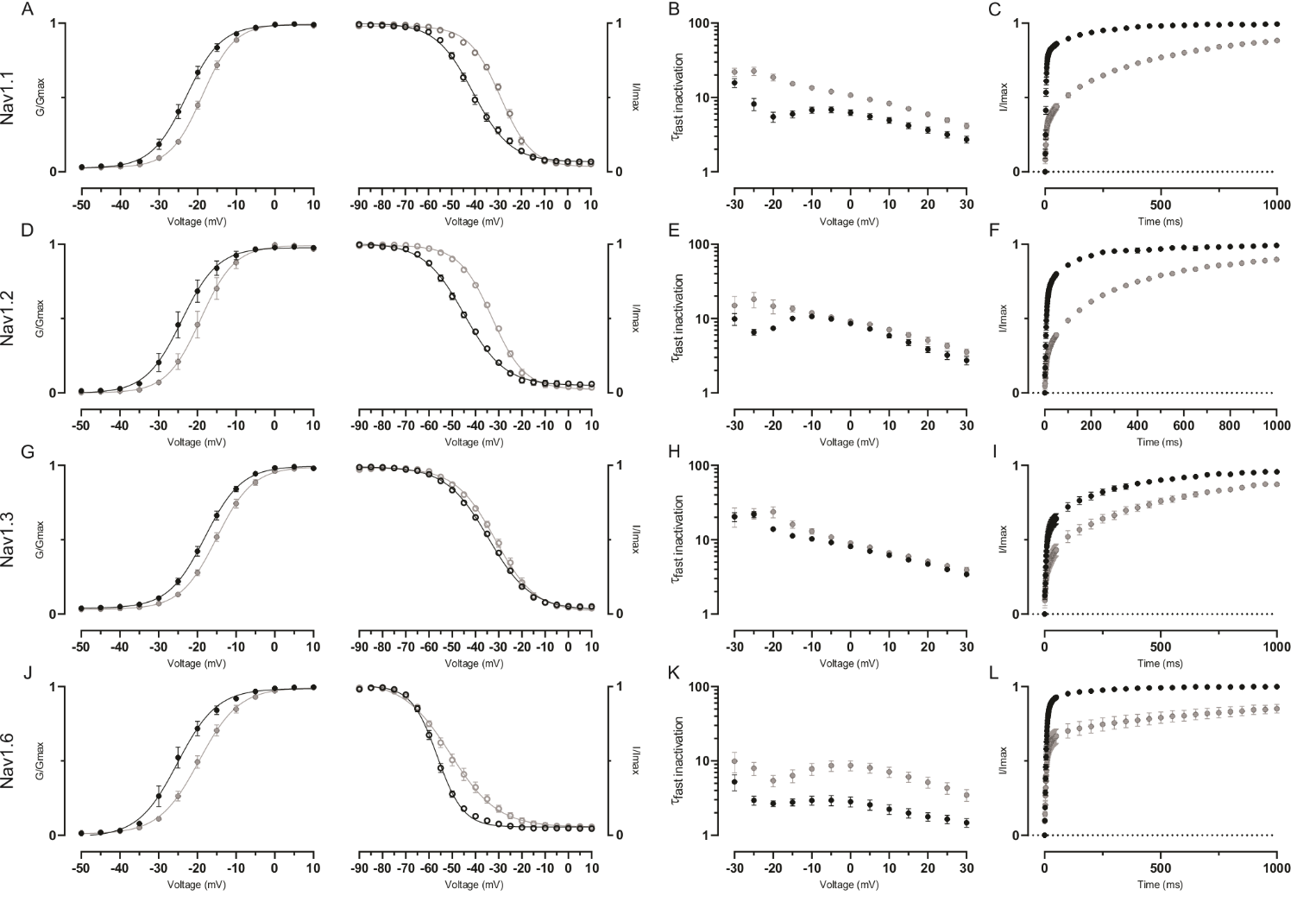
**

**
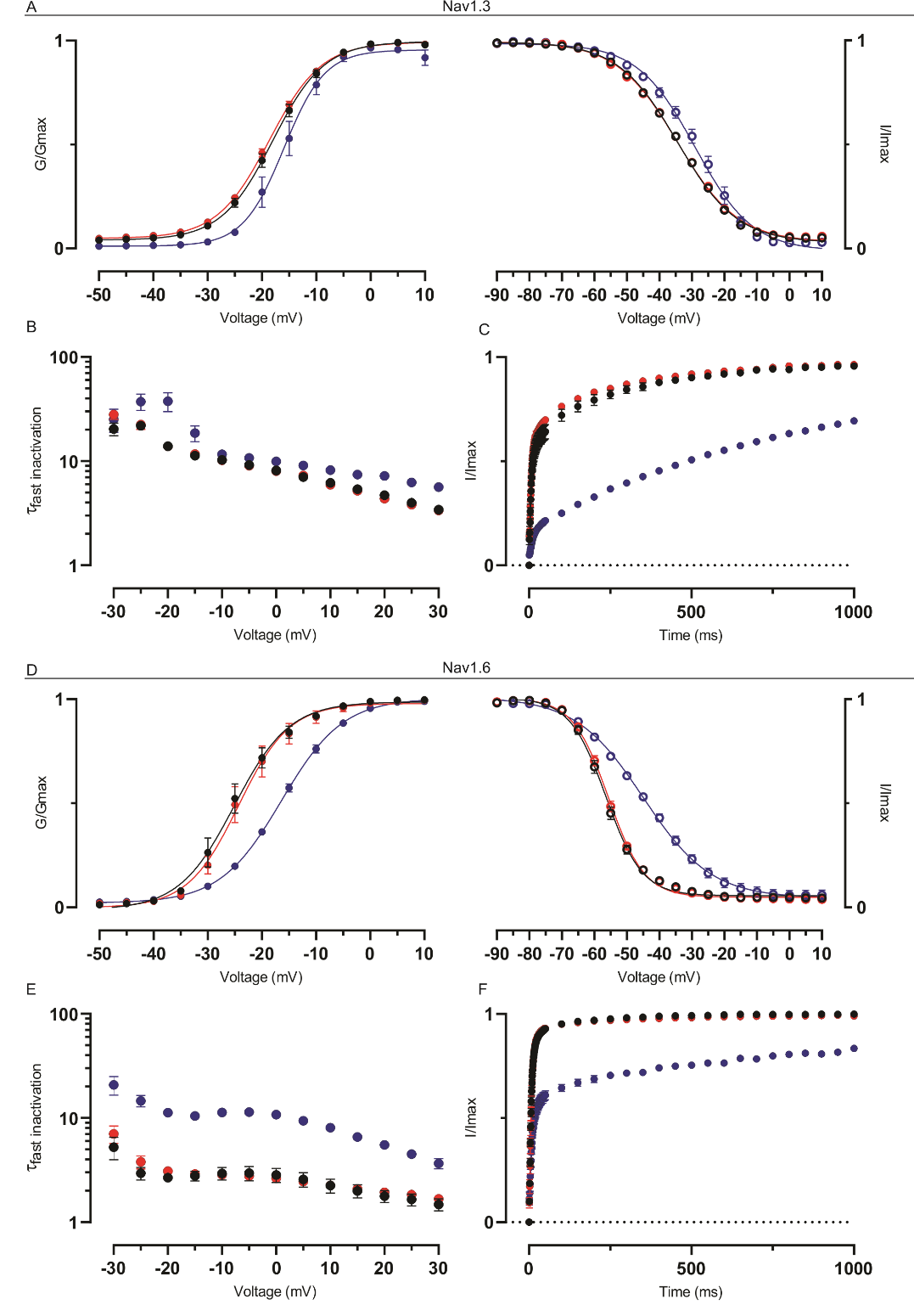
Supplementary figure 3: Effect of β3 mutants on Na_V_1.3 and Na_V_1.6.** **(A)** Normalized conductance-voltage (left) and channel availability (right) curves for Na_V_1.3 in the presence of WT β3 (black), β3^S196*^ (red) or β3^W94^* (blue). **(B)** Inactivation time constants for Na_V_1.3 with WT β3 (black), β3^S196*^ (red) or β3^W94^* (blue). **(C)** Normalized recovery from fast inactivation for Na_V_1.3 with WT β3 (black), β3^S196*^ (red) or β3^W94^* (blue). **(D)** Normalized conductance-voltage (left) and channel availability (right) curves for Na_V_1.6 in the presence of WT β3 (black), β3^S196*^ (red) or β3^W94^* (blue). **(E)** Inactivation time constants for Na_V_1.6 with WT β3 (black), β3^S196*^ (red) or β3^W94^* (blue). **(F)** Normalized recovery from fast inactivation for Na_V_1.6 with WT β3 (black), β3^S196*^ (red) or β3^W94^* (blue). All data is shown as mean ± SEM.
